# Child Crying in Utero

**Published:** 1896-09

**Authors:** W. M. Powell

**Affiliations:** Albany, Texas


For the Texas Medical Journal.

### CHILD CRYING IN UTERO.

BY W. M. POWELL, M. D., ALBANY, TEXAS.

MRS. T. aet. 26. Multipara, sent for me on the night of the 22d inst., thinking she was in labor. I found her nervous and excited. An external examination disclosed the fact that she was not in labor, and I so informed her. Did not ascertain the cause of her nervousness till the following morning, when she told me that herself, sister and husband were sitting out in the moonlight on the lawn when they all distinctly heard the child cry two or three times; this so alarmed her that she immediately took her bed and sent for me. I delivered her on the night of the 24th, of a well developed girl babe, 8½ pounds. No bag of waters presented, nor did any of the liquor amnii escape till after the child was expelled. It was the third time I had delivered her, once of twins. I have no reason to doubt the truthfulness of her statement, corroborated by her sister and husband, who were sitting close by. Having never heard of but one other case of the kind (which I give below), I thought I would report it.

In the Atlanta *Medical and Surgical Journal*, June, 1876, Dr. W. H. Dean, of Woodstock, Ga., reported a case as follows: "I was called on the 7th inst., to see a woman in labor with her fourth child. The membranes ruptured about twenty minutes before I arrived, and a large quantity of liquor amnii was discharged. Made an examination immediately; found cord prolapsed and the head above superior strait. The woman got up to have her bed arranged, and while up the child in utero commenced crying vigorously, so as to be heard all over the room, and if persons had been listening, could have been heard all through the house. This being the first case witnessed in a practice of about thirty years, I concluded to report it."

W. H. DEAN, M. D.

Woodstock, Ga., April 18, 1876.

It will be seen that the difference between Dr. Dean's case and mine, is, that in his case the liquor amnii had escaped, but in mine it had not, and some forty-eight hours had elapsed before my patient was in actual labor.

I report this singular incident to know if any one else has had a similar case, and if possible explain the phenomenon.

